# Supplementary material for: Mixed-state electron ptychography enables sub-angstrom resolution imaging with picometer precision at low dose
Source: Nat Commun. 2020 Jun 12;11:2994. doi: 10.1038/s41467-020-16688-6 (PMC7293311; doi:10.1038/s41467-020-16688-6)
Supplement: Supplementary file 1 — Supplementary Information [file 41467_2020_16688_MOESM1_ESM.pdf]

Supplementary information

Mixed-state electron ptychography enables sub-angstrom resolution imaging with picometer precision at low dose

Chen, et al.

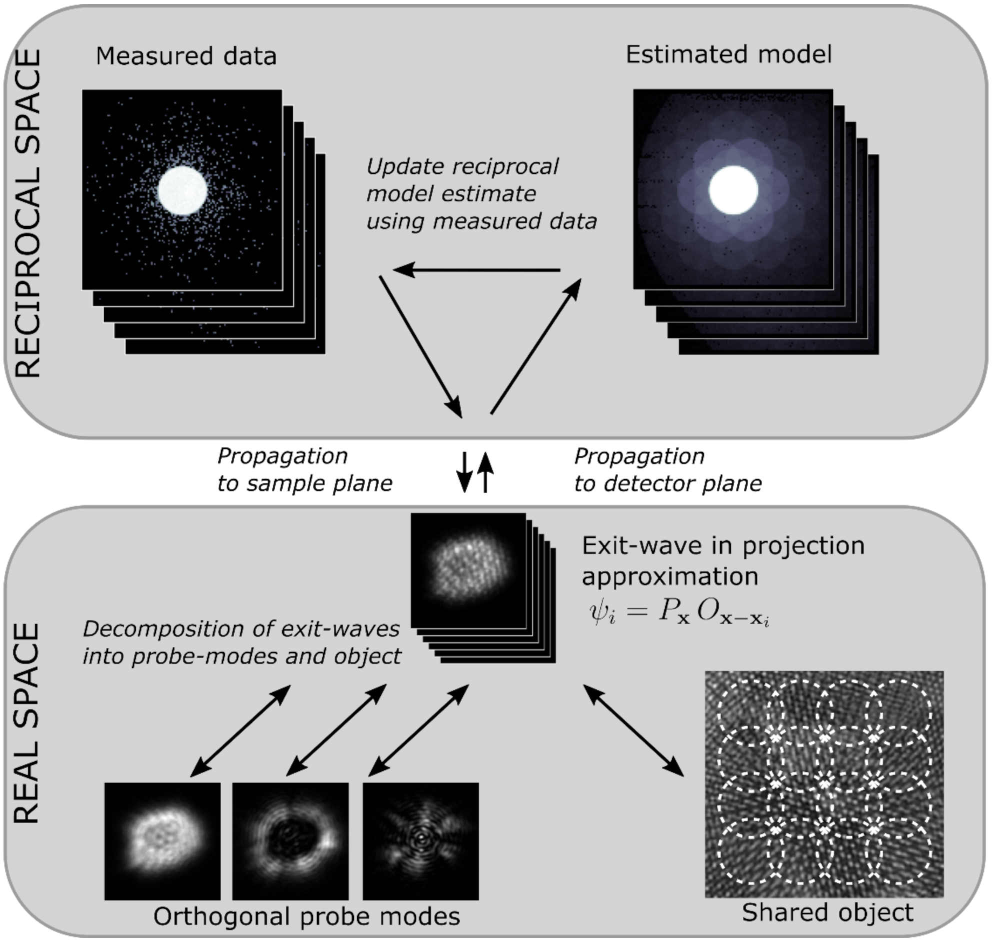

Supplementary Figure 1: A flowchart of mixed-state electron ptychography. The diagram illustrates the working principle of the LSQ-ML ptychography method. At first, initial guesses of the exit-waves computed from the illumination probe modes and complex-valued object function are forward-propagated to the reciprocal space, where they are updated in order to maximize a likelihood function given the measured data. The updated reciprocal models are then back-propagated to the object plane, where a linear least-squares method is used to determine the optimal decomposition into the incoherent probe modes, the shared object function and to refine the estimated scan positions. Finally, new estimates of the exit-waves are calculated from the updated probes and the object and forward-propagated. This procedure is iteratively repeated till convergence.

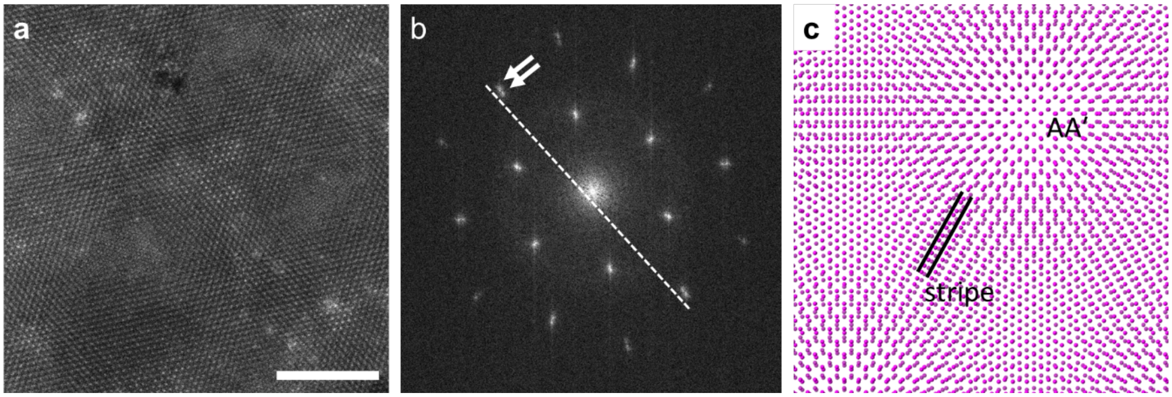

Supplementary Figure 2: ADF image verifying the same orientation of the bilayer sample. a. ADF image from a bilayer area acquired using a conventional annular dark-field detector. Scale bar is 5 nm. **b**. Fourier transform of the image in **a** showing diffraction patterns with splitting spots. **c.** A bilayer $WS_2/MoSe_2$ structural model. Due to 4% lattice mismatch between WS_2 and $MoSe_2$, diffraction spots from bilayer region are at different Fourier frequencies. The splitting spots labeled by white arrows on **b** show the diffraction from different layers. Identical crystal orientation of the two layers is verified by the well aligned peaks towards center spot which is guided by the dashed line. The structural model in **c** shows the hexagonal lattice in the well aligned bilayer (AA′ stacking) region and the stripe shape in the intermediate region.

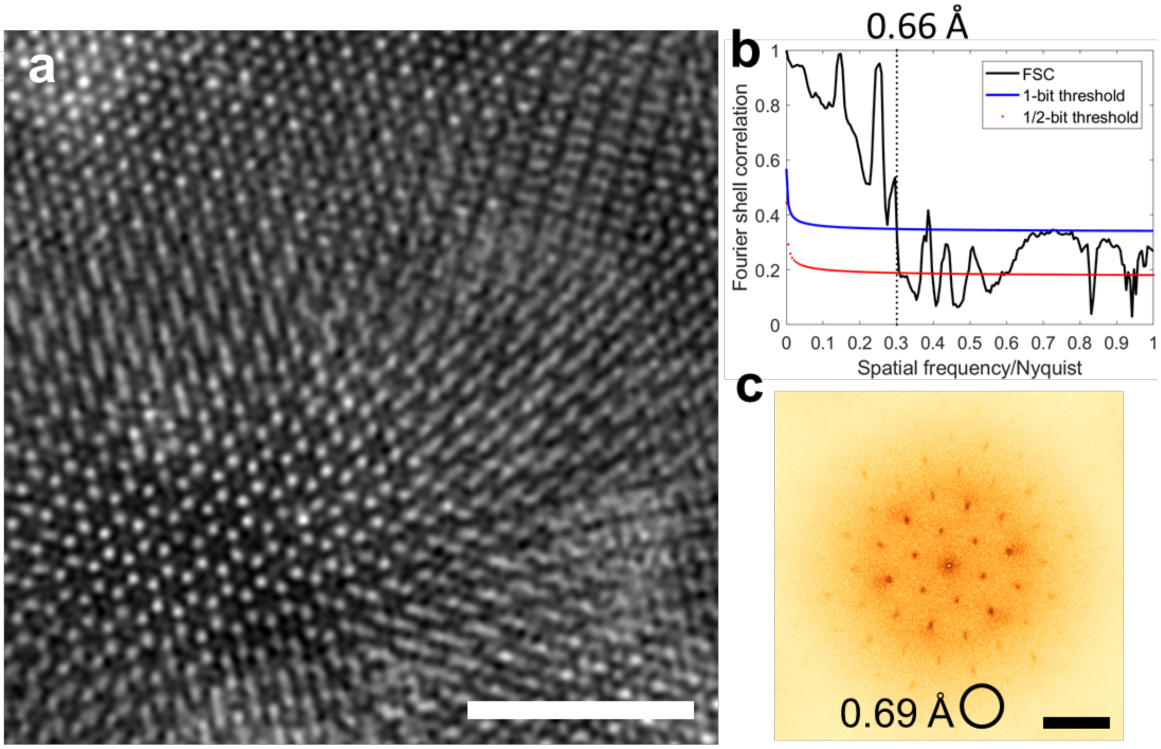

Supplementary Figure 3: Comparison of the spatial resolution determined from two different methods. a. A cropped region of the ptychographic reconstruction shown in the main text Fig. 2**a** from a bilayer $WS_2/MoSe_2$ sample. Scale bar is 2 nm. **b.** The Fourier ring correlation (FRC) of two different scans from the sample area shown in the main text Fig. 2**a**; **c**. The diffractogram of the whole image shown in the main text Fig. 2**a**. Scale bar is 0.7 $Å^{-1}$. The resolution determined from the maximum frequency in the diffractogram and the FRC is very close. Both ½-bit (red line) and 1-bit threshold (blue line) are shown in **b** and 1-bit threshold is chosen to determine the resolution, 0.66 Å.

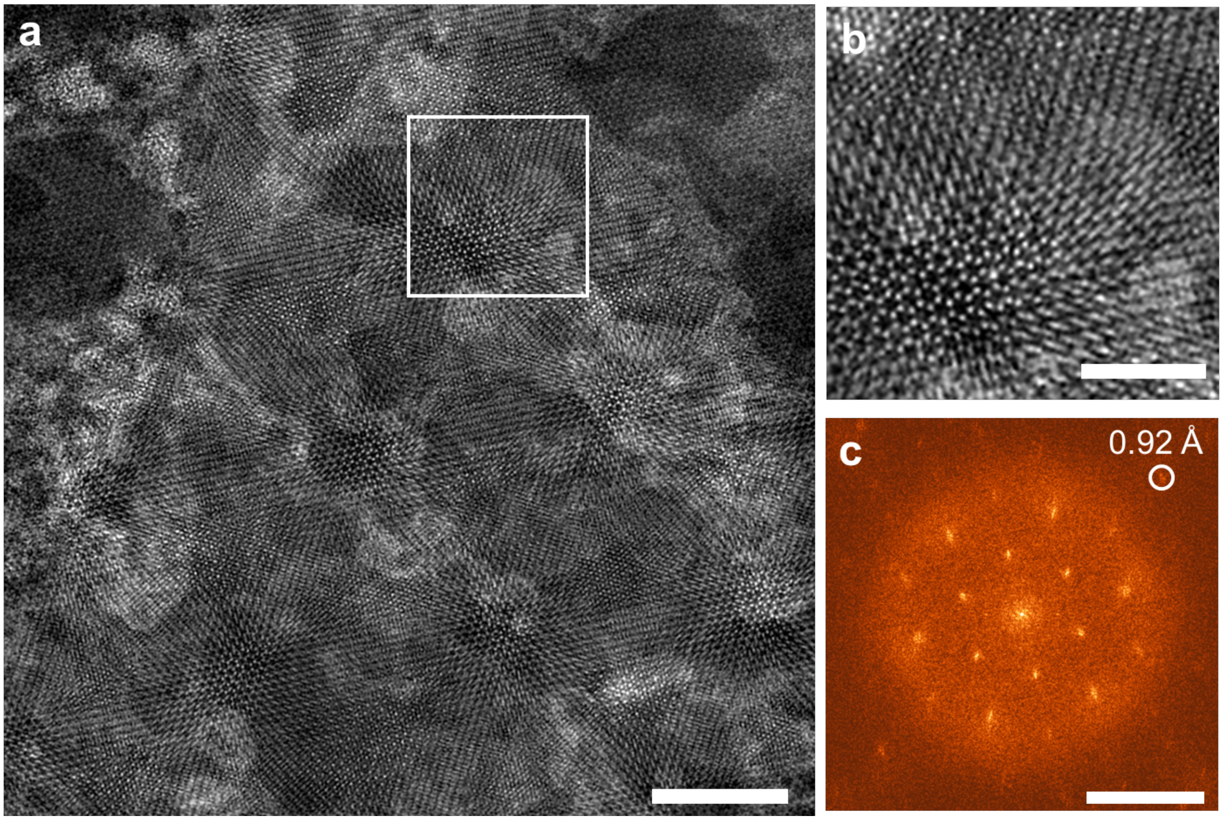

Supplementary Figure 4: Reconstructions from a larger scan step selected from the data used in Fig. 2 in the main text. a. Reconstructed phase image using data with a scan step size of 4.72 Å. Scale bar is 5 nm. **b**. Enlarged region marked on **a**. Scale bar is 2 nm. **c**. A diffractogram of the phase image in **a**. The diffraction spot circulated on **c** corresponds to 0.92 Å in real space. Scale bar is 0.7 $Å^{-1}$. The total number of diffractions of the dataset used for the reconstruction in **a** is 64 × 64. The dose is 4.0×10^3 e $Å^{-2}$.

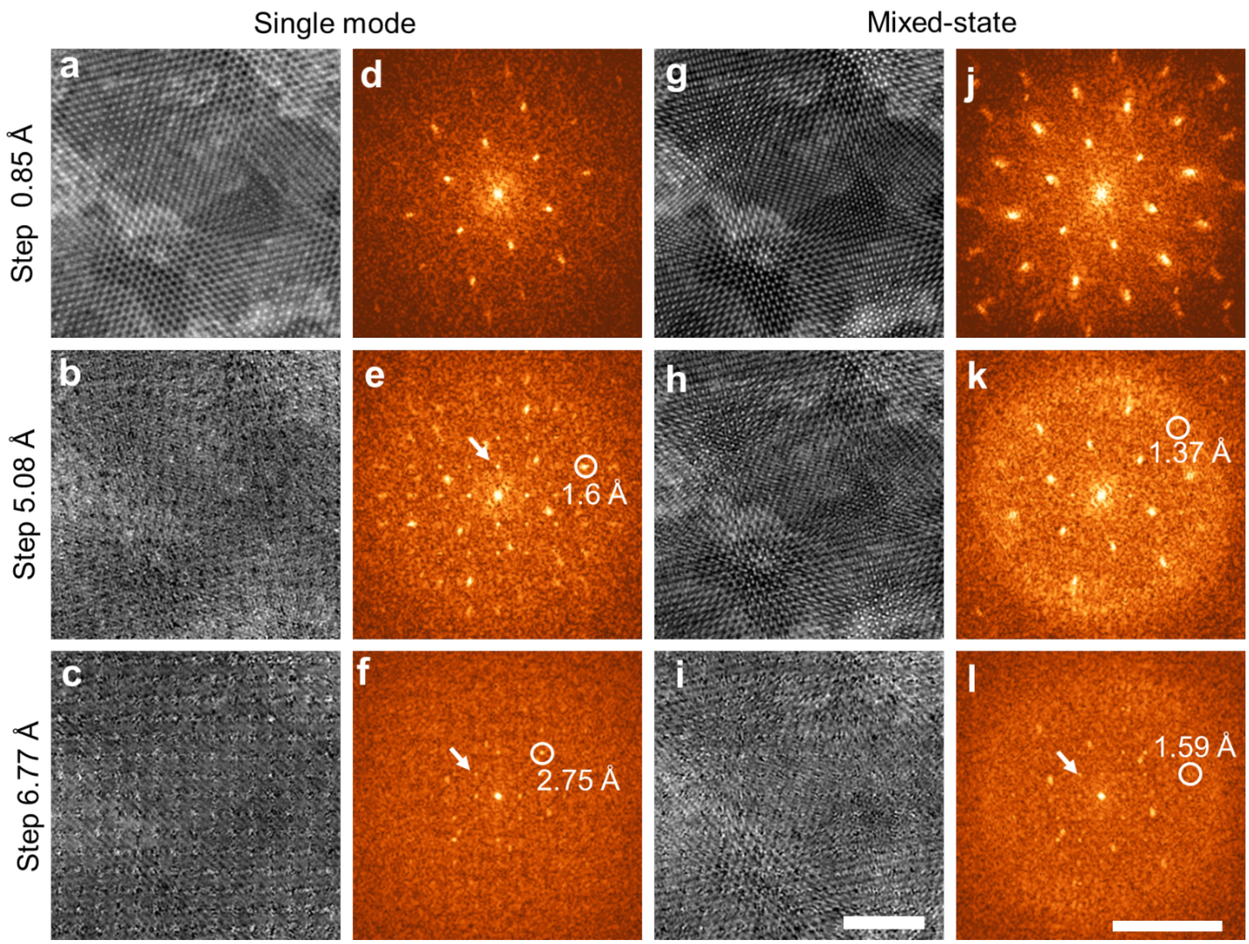

Supplementary Figure 5: Scan step size dependence of single mode and mixed-state ptychography. a-c. Single mode ptychographic reconstructions from datasets using different scan step size; **d-f.** Corresponding diffractogram of **a-c**. **g-i**. Mixed-state reconstructions using two probe modes from datasets using different scan step size; **j-l**. Corresponding diffractogram of **g-i**. Scan step size is 0.85 Å, 5.08 Å and 6.77 Å, and the corresponding overlap ratio is 0.953, 0.718, and 0.624, respectively. The dose is 1.25×10^5 e Å^{-2}, 3470 e Å^{-2}, 1950 e Å^{-2}, respectively. The spots pointed by the white arrows on the diffractograms show the artifacts due to the reconstruction. The single-mode reconstruction can result in an incorrect lattice structure if the overlap is not sufficient, such as in **b** and **c**. Scale bar for real-space images in **a-c** and **g-i** is 3 nm, for diffractograms in **d-f** and **j-l** is 0.7 Å^{-1}.

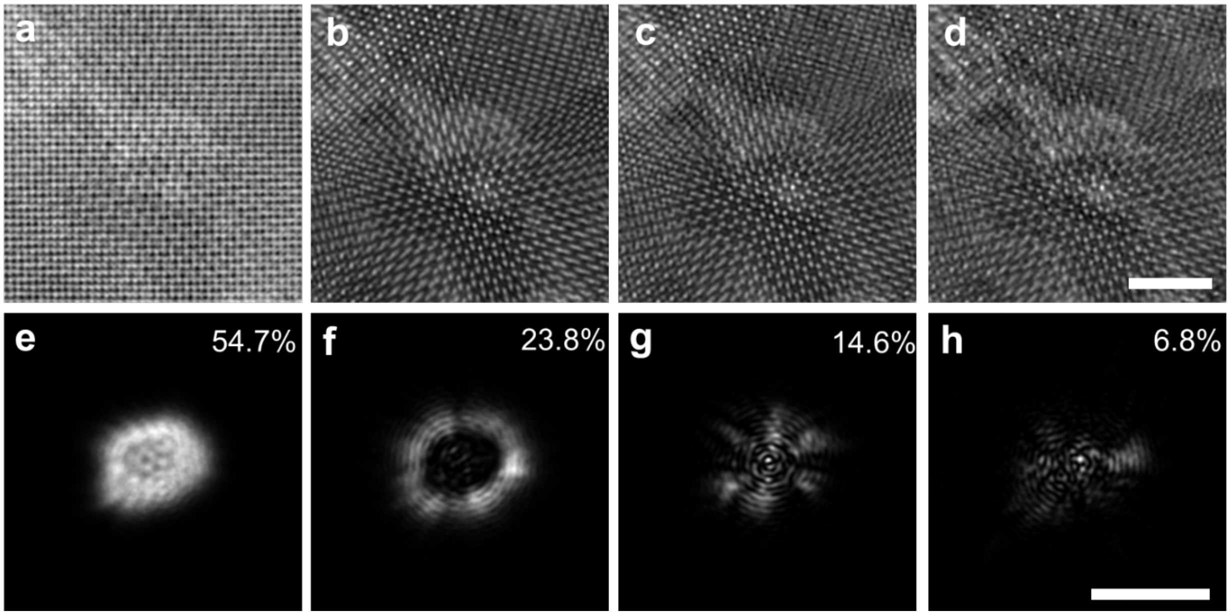

Supplementary Figure 6: Ptychographic reconstructions using different number of probe modes: **a-d.** 1, 2, 4, and 16 modes, respectively. **e-h.** The intensity of the reconstructed illumination function at the object plane from the four-modes mixed-state reconstruction. The scan step size is 1.69 Å, defocus is ~55 nm and the diameter of the probe is ~1.8 nm. The percentage of the power of each mode is denoted on the corresponding probe images. Only about 55% of the illumination power is carried by the dominant coherent mode. The single-mode reconstruction in **a** shows an incorrect lattice structure due to over-simplified reconstruction model without counting for the nonnegligible partial coherence of the probe. Scale bar for **a-d** is 3 nm and for **e-h** is 2 nm.

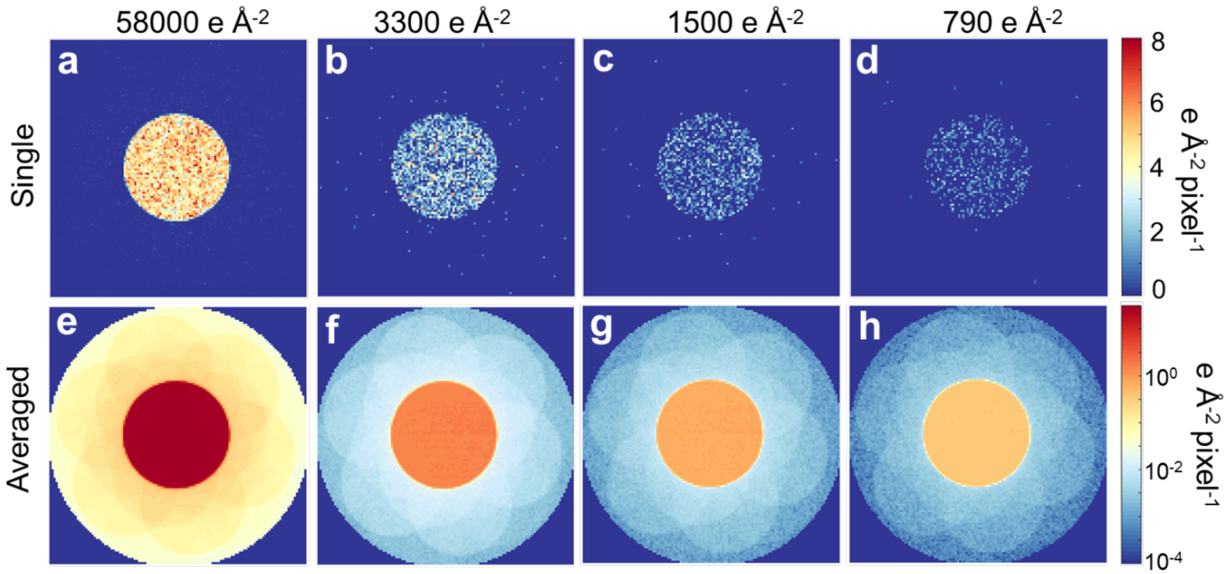

Supplementary Figure 7: Diffraction patterns from datasets acquired using different dose used in main text Fig. 4, single diffraction (first row) and averaged diffractions from 128 × 128 patterns (second row). For the lowest dose, 790 e Å^{-2}, the diffraction contains on average about 500 electrons. Counts of diffraction in **a** was divided by 6 for proper display using the same color bar for **a-d**.

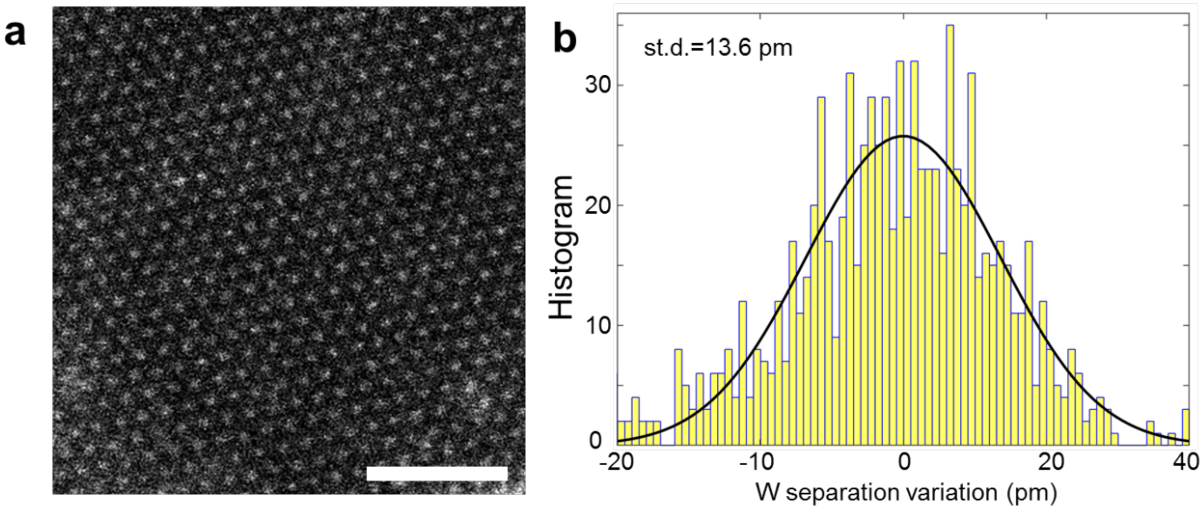

Supplementary Figure 8: Measurement precision of W-W distance from conventional ADF image. a. ADF image from a monolayer WS_2 sample using an illumination dose 4.2 x 10^4 e Å^{-2} with 6 μs per pixel dwell time. Scale bar is 2 nm. **b**. Distribution of W-W atomic separation. Sulfur atoms are invisible, and the atomic positions cannot be determined.
